# Brain tissue properties differentiate between motor and limbic basal ganglia circuits

**DOI:** 10.1002/hbm.22533

**Published:** 2014-04-28

**Authors:** Ettore A. Accolla, Juergen Dukart, Gunther Helms, Nikolaus Weiskopf, Ferath Kherif, Antoine Lutti, Rumana Chowdhury, Stefan Hetzer, John‐Dylan Haynes, Andrea A. Kühn, Bogdan Draganski

**Affiliations:** ^1^ Department of Neurology Charité University Medicine Berlin Berlin Germany; ^2^ LREN Département des Neurosciences Cliniques CHUV Université de Lausanne Lausanne Switzerland; ^3^ Berlin Center for Advanced Neuroimaging Charité Universitätsmedizin Berlin Germany; ^4^ Max Planck Institute for Human Cognitive and Brain Science Leipzig Germany; ^5^ Department of Cognitive Neurology Göttingen University Medical Center Göttingen Germany; ^6^ Wellcome Trust Centre for Neuroimaging London United Kingdom; ^7^ Bernstein Center for Computational Neuroscience Berlin Charité Universitätsmedizin Berlin Germany

**Keywords:** diffusion‐weighted imaging, MT, multiparameter mapping, R2*, voxel‐based quantification

## Abstract

Despite advances in understanding basic organizational principles of the human basal ganglia, accurate in vivo assessment of their anatomical properties is essential to improve early diagnosis in disorders with corticosubcortical pathology and optimize target planning in deep brain stimulation. Main goal of this study was the detailed topological characterization of limbic, associative, and motor subdivisions of the subthalamic nucleus (STN) in relation to corresponding corticosubcortical circuits. To this aim, we used magnetic resonance imaging and investigated independently anatomical connectivity via white matter tracts next to brain tissue properties. On the basis of probabilistic diffusion tractography we identified STN subregions with predominantly motor, associative, and limbic connectivity. We then computed for each of the nonoverlapping STN subregions the covariance between local brain tissue properties and the rest of the brain using high‐resolution maps of magnetization transfer (MT) saturation and longitudinal (R1) and transverse relaxation rate (R2*). The demonstrated spatial distribution pattern of covariance between brain tissue properties linked to myelin (R1 and MT) and iron (R2*) content clearly segregates between motor and limbic basal ganglia circuits. We interpret the demonstrated covariance pattern as evidence for shared tissue properties within a functional circuit, which is closely linked to its function. Our findings open new possibilities for investigation of changes in the established covariance pattern aiming at accurate diagnosis of basal ganglia disorders and prediction of treatment outcome. *Hum Brain Mapp 35:5083–5092, 2014*. © **2014 The Authors. Human Brain Mapping Published by Wiley Periodicals, Inc.**

## INTRODUCTION

The steadily growing number of patients treated with deep brain stimulation of basal ganglia structures motivates further in‐depth investigation of the anatomy and function of the corticosubcortical circuits. Exemplified on the well‐established stimulation of the subthalamic nucleus (STN) for treatment of motor symptoms in idiopathic Parkinson's disease, we acknowledge the emergence of behavioral and cognitive side effects related to the specific site of stimulation [Mallet et al., 2007; Smeding et al., 2006].

Experimental and clinical findings confirm the existence of a certain degree of topological specialization within the basal ganglia corresponding to their implication in motor, associative, and limbic functions. As part of the corticosubcortical circuitry the STN is thought to follow this organizational principle in such a way that motivational and emotional content are attributed to anterior portions and motor control to its posterior part [Hamani et al., 2004; Krack et al., 2010; Mallet et al., 2007; Temel et al., 2005; York et al., 2009]. However, neither invasive animal nor in vivo human imaging studies were able to provide strong empiric evidence for its tripartite organization [for review, see Keuken et al., 2012] or for strict anatomical borders among functional subregions [Haynes and Haber, 2013].

In humans, the functional anatomy of basal ganglia can be studied in vivo using magnetic resonance imaging (MRI) under the assumption of topological correspondence between anatomical connectivity patterns and functional subdivisions. Topology estimates of STN anatomical projections based on MRI sensitive to water diffusion suggested a tripartite organization of the STN; however, this notion was confirmed only in 14 of 24 STNs studied [Lambert et al., 2012]. A recent report using similar methodology reported the existence of a posterior–anterior gradient in STN motor projections rather than clearly delineated partitions [Brunenberg et al., 2012].

Alternatively, anatomical connectivity patterns in the brain can be studied on the basis of MRI‐derived regional volume covariance between putatively connected areas with common maturation pathways [Mechelli et al., 2005; Soriano‐Mas et al., 2013]. Structural covariance studies [Lerch et al., 2006; Schmitt et al., 2010] demonstrated patterns of anatomical connectivity, previously established in diffusion‐tensor imaging experiments. We reason that similarly, anatomical connectivity can be studied by testing for regional covariance of brain tissue properties. Recent studies demonstrated the feasibility of quantitative measurements of the longitudinal relaxation rate (R1 = 1/T1), magnetization transfer (MT) saturation [Helms et al., 2008], and effective transverse relaxation rate (R2* = 1/T2*), which are indicative for local free tissue water, myelin, and iron content. The direct correspondence between MT, R1, and R2* values and the underlying brain tissue properties is not yet demonstrated. According to the underlying biophysical model, R1 captures not only free water molecules but also macromolecules, thus sensitive to myelin, and is further influenced by iron content [Rooney et al., 2007]. MT saturation is indicative for myelin content and is in principle not affected by T1 effects [Helms et al., 2008, 2009]. The linear dependency between the transverse relaxation rate R2* and iron content has been validated with postmortem measurements [Langkammer et al., 2010].

Considering previous reports about gradients of iron content in the STN spatially overlapping with functional subregions [Dormont et al., 2004; Massey et al., 2012], we hypothesized that local tissue properties within motor, associative, and limbic STN subregions will covary with corresponding subregions within basal ganglia circuits.

Here, we present a detailed analysis of the regional covariance of myelin and iron content within limbic and motor basal ganglia circuits that combines different anatomical MRI modalities—probabilistic diffusion tractography and quantitative multiparameter mapping within the well‐established framework of voxel‐based quantification, VBQ [Dick et al., 2012; Draganski et al., 2011; Lambert et al., 2013; Sereno et al., 2013].

## MATERIALS AND METHODS

### Participants

Thirteen subjects (six females, age range: 40–72 years; mean 50.6, standard deviation: 10.9 years) underwent diffusion‐weighted imaging and quantitative multiparametric brain imaging on a 3‐T whole‐body MRI system (Magnetom TIM Trio, Siemens Healthcare, Erlangen, Germany) using a 32‐channel radiofrequency (RF) head receive coil and RF body transmit coil. Multiparametric brain imaging data were pooled together with data from a separate cohort to a total of 101 healthy subjects (64 females, age range: 40–71 years; mean: 59.6, standard deviation: 16.5 years), previously acquired with the same protocol to study the effects of healthy aging [Chowdhury et al., 2013]. Subjects gave written informed consent for use of their anonymized data in studies led by the responsible investigator. Local ethics committees approved all experimental protocols.

### Magnetic Resonance Imaging

The whole‐brain quantitative MR imaging (101 subjects) consisted of 3D multiecho FLASH datasets with predominantly proton density weighting (PDw; repetition time, TR = 23.7 ms, flip angle *α* = 6°), T1 weighting (T1w; TR/*α* = 18.7 ms/20°), and MT weighting (MTw; TR/*α* = 23.7 ms/6°) contrast according to the previously published protocol [Weiskopf et al., 2013]. Multiple gradient echoes were acquired with alternating readout polarity at six equidistant echo times (TE) between 2.2 and 14.7 ms for the T1w and MTw acquisitions and at eight equidistant TE between 2.2 and 19.7 ms for the PDw acquisition. We used the following acquisition parameters: 1 mm × 1 mm × 1 mm voxel size, field of view (FOV) 256 mm × 240 mm × 176 mm, matrix 256 × 240 × 176, GRAPPA factor 2 in phase‐encoding (PE) direction, 6/8 partial Fourier in partition direction, and nonselective RF excitation. For correcting inhomogeneous RF excitation effects we map the distribution of the RF field (B1+) over the brain. The B1+ maps are acquired using 3D echoplanar imaging spin‐echo (SE)/stimulated echo (STE) method described by Lutti et al. [2010, 2012]. The following acquisition parameters were used for the acquisition of the B1+ data: FOV 256 mm × 192 mm × 192 mm, matrix 64 × 48 × 48, and TR = 500 ms. The B1+ maps were corrected for EPI image distortions and off‐resonance effects using a standard B0 map according to the published protocol [Lutti et al., 2012].

The diffusion‐weighted imaging protocol was performed with the following parameters: TE = 80 ms, TR 8,300 ms, acquisition matrix 128 × 128 voxels, 74 axial slices, yielding voxel size of 1.7 mm × 1.7 mm × 1.7mm, BW = 2,003 Hz/pixel, diffusion weighting at a high *b* = 1,000 s/mm^2^ along 60 directions, and six reference volumes at zero *b*‐value acquired one every 10th high *b*‐value acquisition.

### Data Processing

Image processing was performed with the freely available Statistical Parametric Mapping software (SPM8; Wellcome Trust Centre for Neuroimaging, London, UK, http://www.fil.ion.ucl.ac.uk/spm/software/), running under Matlab 7 (Mathworks, Sherborn, MA). Probabilistic diffusion tractography was performed with the FDT diffusion toolbox in the framework of FSL [Behrens et al., 2007].

#### Multiparameter maps computation

The computation of the longitudinal relaxation rate (R1 = 1/T1), MT saturation, and effective transverse relaxation rate (R2* = 1/T2*) parameter maps was performed as described previously in the context of VBQ [Draganski et al., 2011; Weiskopf et al., 2013]. For optimal delineation of basal ganglia structures [Helms et al., 2009] the MT maps were classified into gray matter (GM), white matter (WM), and cerebrospinal fluid using Gaussian mixture model within the “unified segmentation” framework [Ashburner and Friston, 2005]. The MT, R1, and R2* parameter maps were spatially registered to standard MNI space using the subject‐specific diffeomorphic estimates from the DARTEL procedure implemented in SPM8 [Ashburner, 2007] after weighting with the corresponding tissue probability map as described previously [Draganski et al., 2011]. For statistical analysis, we smooth the data with an isotropic Gaussian kernel of 6 mm full‐width‐at‐half‐maximum.

#### Probabilistic diffusion tractography

To allow bias‐free definition of seed and target areas we performed supervised detection and labeling of the STN in subject‐specific native space. We used an approach based on affine transformation of the surface‐based digitalized Morel histological atlas [Krauth et al., 2010] for exact match to the MNI space [Schönecker et al., 2009]. The inverse of the subject‐specific diffeomorphic estimates from the DARTEL procedure in the previous step was applied to the Morel's atlas to spatially register it in the individual's native space. The same approach was used to build target masks of motor, associative, and limbic cortical targets. Motor areas (primary motor cortex—M1 and supplementary motor area—SMA), associative prefrontal areas (superior, middle, and inferior frontal gyri), orbitofrontal cortex, and anterior cingulate cortex (ACC) were labeled according to the Harvard–Oxford atlas [Desikan et al., 2006], whereas medial temporal structures (hippocampus and amygdala) were labeled using the Juelich atlas [Eickhoff et al., 2005].

Probabilistic tractography was performed in subject‐specific native space after affine registration and correcting for eddy currents using the default settings in FSL bedpostx with the following parameters: 5,000 originating tracts per voxel, curvature 0.2, and step length 0.5. Distributions of diffusion parameters were estimated at each voxel to model the directions of up to two tensors per voxel [Behrens et al., 2007]. We use the option “classification targets” of probtrackx to obtain per each target a map of the connectivity of the STN where for each STN voxel we compute the number of tracts (on a total of 5,000) reaching the target.

#### STN connectivity maps

Three STN connectivity maps were built for each side: (i) “motor” map—STN probabilistic connectivity distribution to primary motor cortex and the supplementary motor cortex; (ii) “prefrontal associative”—connectivity to prefrontal cortex including superior, middle, and inferior frontal gyri; and (iii) “limbic” map— connectivity distribution to ACC, hippocampus, amygdala, and orbitofrontal cortex including medial, anterior, posterior, and lateral orbitofrontal cortex, but not the medial surface of gyrus rectus. All maps were spatially registered to MNI space for further analysis by applying the spatial registration estimates from the previous step. To obtain a Gaussian distribution and to minimize variability across subjects, connectivity values were first normalized (*z*‐score). We then built exclusive motor, associative, and limbic STN connectivity maps by assigning at the voxel level the highest probabilistic value to a particular area on the “winner‐takes‐all” principle after subtraction of probabilistic connectivity values from the two other subregions.

### Statistical Analysis

The exclusive masks obtained from diffusion tractography (13 subjects) were used to delineate motor, associative, and limbic STN in multiparameter maps (101 subjects). This allowed for extraction of mean MT, R1, and R2* values from each STN subarea in each subject to be used for further analysis.

Aiming at investigating differential tissue properties underlying STN functional specialization, we performed separate analyses of variance (ANOVAs) for each quantitative map values (MT, R1, and R2*) extracted from STN subareas, followed by *t*‐tests (motor vs. associative, associative vs. limbic, and limbic vs. motor; significance set at *P* < 0.05).

To test the hypothesis of brain tissue property covariance within functional basal ganglia circuits, we computed voxel‐wise correlations between parameter values extracted from motor, associative, or limbic STNs and the rest of the brain. The usage of different datasets improves the generalizability of results, as radically different methods are used to delineate functional areas in STN (diffusion‐weighted imaging) and in the rest of the basal ganglia (covariance of relaxometry‐based multiparameter maps). We used a multiple linear regression model as implemented in the general linear model framework of SPM. Age and gender were included as additional variables in the design matrix. All parameter data (MT, R1, and R2*) were included in the same model after concatenation using a mass‐univariate approach with single block‐diagonal design matrix structure. This ensures the same beta‐parameter estimates as in the separate component analyses. Different variance components were estimated for each block of data separately using REML nonsphericity estimations [Friston et al., 2005]. Voxel‐based two‐tailed *T*‐statistics were computed to detect regional correlation for each parameter map regarding motor, associative, and limbic STN regional maps separately. Statistical thresholds were applied at *P* < 0.05 after family‐wise error (FWE) correction for multiple comparisons over the whole volume of the GM/WM mask. Trends were assessed by using an auxiliary uncorrected voxel threshold of *P* < 0.001 [Friston et al., 1994]. After a whole brain analysis showing absence of cortical regions covarying with the STN we restricted the search volume to the extent of subcortical structures—basal ganglia and thalamus (STN excluded) —using an inclusion mask derived from the Basal Ganglia Human Area Template [BGHAT—Janey Prodoehl et al., 2008].

## RESULTS

The semiautomated STN identification in native space was visually inspected for gross misalignments. The average seed volume of the STN was 173.84 ± 19.99 mm^3^.

### STN Connectivity—Probabilistic Diffusion Tractography

**Figure 1:**
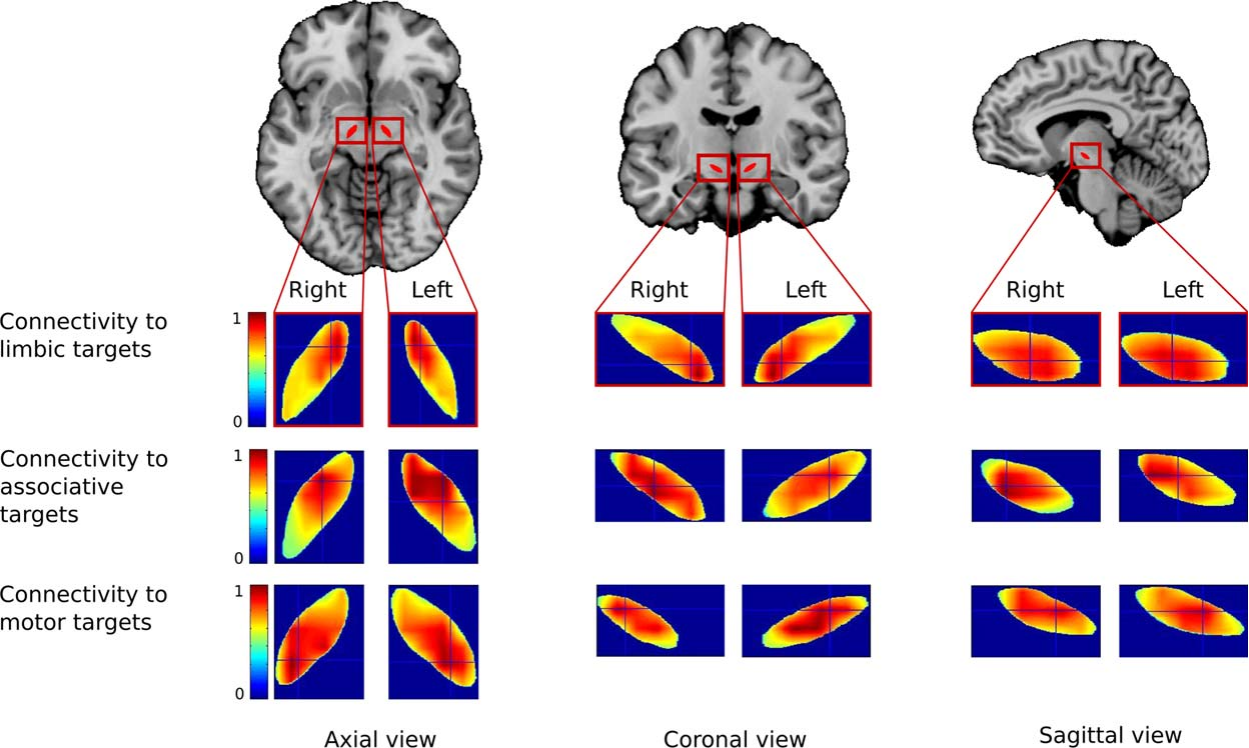
Representation of averaged (*n* = 13) subthalamic nucleus—STN, probabilistic connectivity gradients for motor, associative, and limbic cortical areas. Hot colors represent high connectivity probability, and cold colors represent low probability. Axial (left panel), coronal (middle panel), and sagittal view (right panel) of connectivity to limbic (top row), associative (middle row), and motor (bottom row) cortical areas. [Color figure can be viewed in the online issue, which is available at http://wileyonlinelibrary.com.]

The probabilistic diffusion tractography conducted on 13 subjects demonstrated STN connectivity patterns with distinct gradients in the posterolateral to anteromedial direction (Fig. 1). The connectivity to motor areas was higher in the posterolateral region of the nucleus, progressively decreasing along medial, inferior, and anterior regions. The opposite was observed for connectivity to limbic areas with a maximum reached in the anterior tip of the nucleus, while connectivity to associative areas was highest in the centroinferior portion of the nucleus. STN segmentation was consistent with this pattern, with most posterosuperior regions labeled as motor, central as associative, and the most anteromedial as limbic.

#### Multiparameter values of STN subregions

Mean values of multiparameter maps in the limbic, associative, and motor parts of the STN differed significantly across the 101 subjects' scans (paired *t*‐tests, Table 1). R2* values, reflecting iron deposition, were highest in the anterior (limbic) STN, decreasing posteriorly in the associative region and furthermore in the motor region. An opposite profile was observed for R1, sensitive to both water and myelin content, highest in the motor and lowest in the limbic portions. The highest MT values were observed in the associative STN, yet higher in the motor than in the limbic subregions. The ANOVAs testing for differences between limbic, associative, and motor subregions showed significant results for all three modalities (*P* < 0.05). *t*‐Tests confirmed the significance of differences between associative and limbic next to differences between motor and limbic subregions (*P* < 0.05). The summary of statistical results is presented in Table 1.

**Table 1:** Brain tissue properties within STN subregions

	MT		R1		R2*	
Limbic	1.1552	(0.1018)	0.866	(0.170)	29.6	(0.75)
Limbic vs. associative	<0.001		<0.001		0.017	
Associative	1.3050	(0.1128)	0.921	(0.177)	29.2	(0.63)
Associative vs. motor	0.2980		0.1050		0.057	
Motor	1.3026	(0.1150)	0.923	(0.178)	28.8	(0.6)
Motor vs. limbic	<0.001		<0.001		0.002	

Mean (SD) magnetization transfer (MT) saturation (in %), longitudinal relaxation rate R1 (1/T1) (in s^−1^), and transversal relaxation rate R2* (in s^−1^) in limbic, associative, and motor parts of the subthalamic nucleus—STN (*n* = 101). *P*‐values correspond to paired *t*‐test between the multiparameter maps values in limbic, associative, and motor STN subregions.

### VBQ Covariance Analysis

#### Tripartite STN

R2* and MT maps in the motor STN showed the highest covariance with thalamus, posterior putamen, and pallidum, as well as with a small central area in the head of the right caudate nucleus. R1 and MT maps in the associative STN covaried with centrodorsal pallidum, both pars externa (GPe) and pars interna (GPi). R2* maps in the limbic STN covaried with the anteroventral internal and external pallidum (Table 2).

**Table 2:** Covariance analysis for multiparameter maps values extracted from a tripartite (motor, associative, and limbic) or bipartite (motor and limbic/associative) STN

		Coordinates (*x,y,z*)			*Z*‐score	*P*
Tripartite STN						
Motor	MT	−15	−14	3	3.55	<0.001*
		−14	−21	−6	3.50	
		16	−15	3	3.27	
	R1	—	—	—	—	—
	R2*	24	−12	−3	4.77	<0.05
Associative	MT	−10	−2	2	4.18	<0.001*
		12	−2	2	4.06	
	R1	16	−2	6	Inf	<0.05
		6	−6	8	6.75	
		−10	−2	2	6.94	
	R2*	—	—	—	—	—
Limbic	R1	—	—	—	—	—
	MT	—	—	—	—	—
	R2*	−12	1	−6	4.55	<0.05
		12	1	−6	4.49	
		16	−2	−9	4.12	
Bipartite STN						
Motor	MT	−22	−14	−3	6.99	<0.05
		−15	−17	2	6.96	
		18	−8	−3	6.86	
	R1	−14	−18	0	Inf	<0.05
		−14	−21	−6	7.66	
		8	−20	0	7.19	
		−20	−9	−3	7.17	
		−20	−24	−6	5.78	
		24	−14	−1	5.37	
	R2*	20	−9	4	Inf	<0.05
Limbic/Associative	MT	10	−2	−3	7.64	<0.05
		−9	−2	−4	6.9	
		−32	−11	9	4.83	
	R1	8	4	−7	5.15	<0.05
		10	−2	−4	5.99	
		−4	4	−6	5.04	
	R2*	10	−2	−4	Inf	<0.05
		−15	−2	−9	Inf	
		−4	3	2	5.44	
		6	3	2	4.92	

Coordinates (*x,y,z*) represent the centers of voxel clusters covarying with values of MT, R1, and R2* extracted from STN subregions. Covariance was considered significant for *P* < 0.05 after family‐wise error correction for multiple comparisons (*uncorrected).

Given the relatively small size of the STN (a total of 36 voxels per hemisphere) we aimed at investigating the robustness of our inferences and repeated the analysis seeding from a bipartite STN. We defined subsequently a “limbic–associative” (=limbic + associative) and a “motor” STN.

#### Bipartite STN

**Figure 2:**
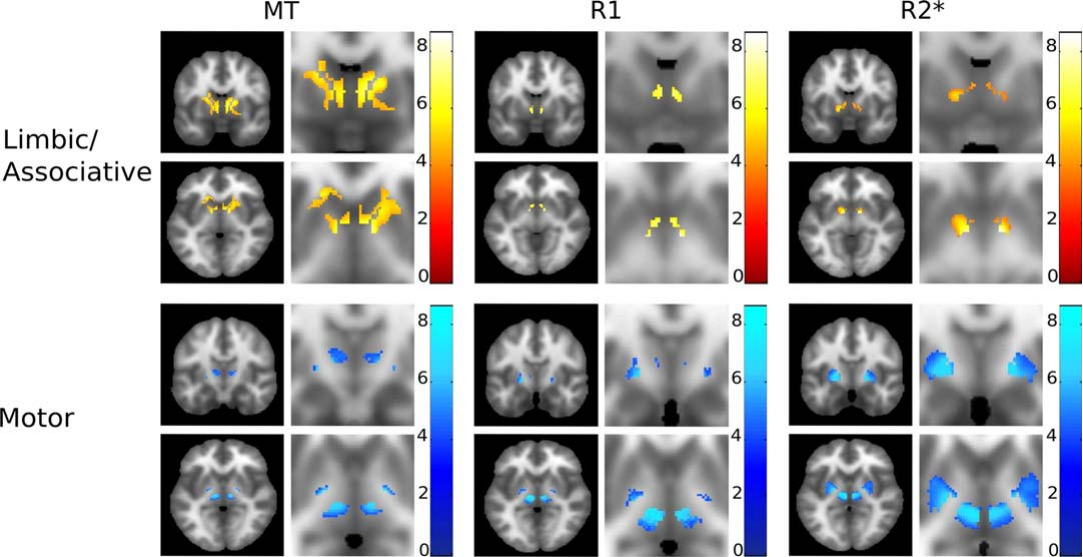
Covariance of multiparameter maps between motor and limbic/associative STN subregions and corresponding areas in basal ganglia. Statistical parametric maps of significant magnetization transfer (MT) saturation, longitudinal relaxation rate R1 (1/T1), and effective transversal relaxation rate (R2*) covariance with limbic/associative (yellow) and motor STN (blue) are superimposed on averaged MT saturation maps of all study participants. Results are presented after *P* < 0.05 family‐wise error correction for multiple comparisons. [Color figure can be viewed in the online issue, which is available at http://wileyonlinelibrary.com.]

R2* maps in the motor STN showed the highest covariance with posterior putamen, pallidum, and in a wide anterior thalamic area encompassing the anterior, mediodorsal, laterodorsal, ventral anterior, and ventral lateral nuclei (Table 2, Fig. 2). MT and R1 maps in the motor STN demonstrated similar covariance pattern distributed over the ventral anterior and lateral, as well as laterodorsal thalamic nuclei, GPi (MT and R1), and left GPe (only R1).

MT maps in the limbic/associative STN covaried with the dorsal, anterior, and ventral caudate, anterior putamen, and ventral pallidum (GPi and GPe). R1 maps in the limbic STN covaried with R1 values in the ventral–anterior caudate. The R2* maps of the limbic STN showed significant covariance with the ventral GPi and GPe.

## DISCUSSION

Here, we demonstrate in vivo that STN subregions can be characterized not only by specific anatomical projections but also by differential brain tissue properties measured with MRI. More importantly, the covariance of iron and myelin content between STN subregions and corresponding areas within basal ganglia circuits is interpreted as evidence for shared tissue properties within specific functional subcortical networks. Our findings open new possibilities to study the anatomical organization of the STN as part of the corticosubcortical circuitry and have potential impact on attempts to define intraindividually the optimal target for deep brain stimulation.

### STN Connectivity—Probabilistic Diffusion Tractography

Taking advantage of high‐resolution diffusion‐weighted sequences, we demonstrate gradients of connectivity to motor, associative, and limbic cortical regions, spanning along the entire length of the STN. Here, we did not observe clear‐cut boundaries among functional subregions. Corroborating the results from a recent tracing study in non‐human primates investigating the patterns of STN connectivity, we interpret our findings as evidence for spatial overlap of convergent projections [Haynes and Haber, 2013]. Considering the focus of our study on the covariance analysis of brain tissue properties we base our inferences on nonoverlapping STN partitions to provide clear‐cut, although admittedly simplified, interpretation of shared tissue properties within basal ganglia circuits.

### Brain Tissue Properties in STN Subregions

Main finding of our study is the in vivo evidence for differential tissue properties within the STN consistent with lower myelin and higher iron content in limbic areas compared with motor and associative functional regions, which is in line with previous imaging and postmortem studies [Dormont et al., 2004; Massey et al., 2012; Schäfer et al., 2012]. We interpret our findings showing regional specificity of MT and R1 within STN as evidence for the presence of densely packed myelinated axons in the motor and associative part of STN, which is known from studies in non‐human primates [Mathai et al., 2013]. On the other hand, the specific spatial pattern of STN R2* values, indicative for iron content, most likely originates from ferritin deposits in oligodendrocytes particularly owing to their role in the myelin synthesis [Connor and Menzies, 1996; Fukunaga et al., 2010].

### Covariance of Tissue Properties

**Figure 3:**
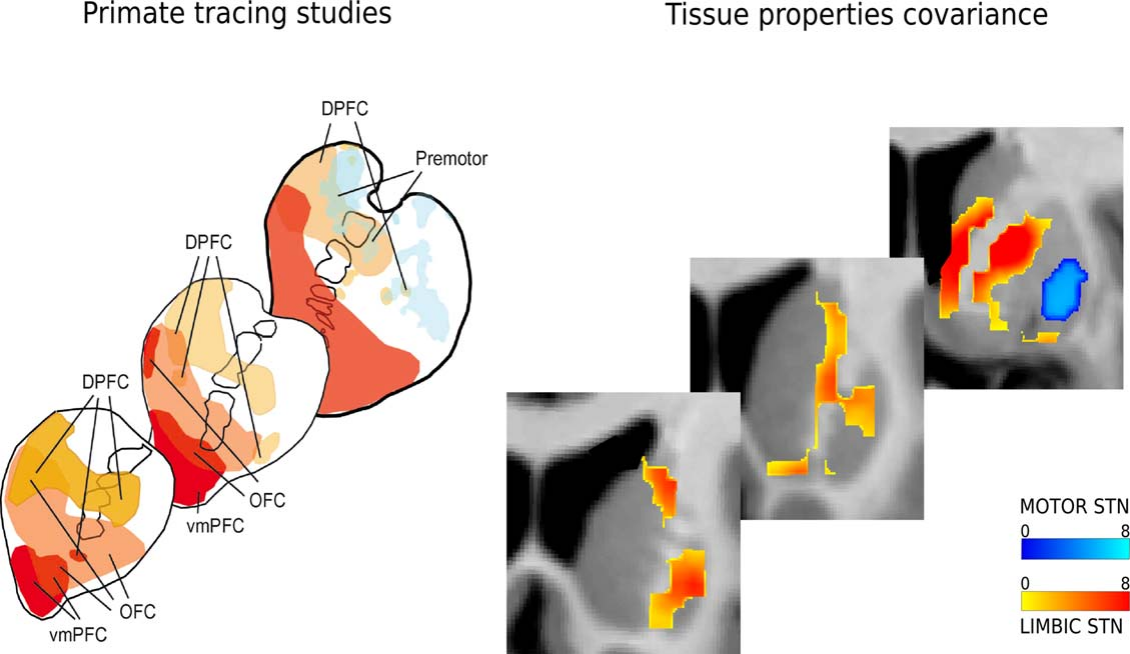
Panel right: Representation of tissue property covariance results within the striatum (putamen and caudate nucleus). Statistical parametric maps of tissue property covariance between striatum and limbic/associative (yellow) or motor (blue) STN. Magnetization transfer (MT) saturation maps best captures limbic/associative covariance (yellow), while effective transverse relaxation rate, R2*, is more relevant for motor covariance (blue). Panel left: Corresponding slices from tracing studies in non‐human primates (from Haber, 2010, with permission). OFC, orbitofrontal cortex; DPFC, dorsolateral prefrontal cortex; vmPFC, ventromedial prefrontal cortex. [Color figure can be viewed in the online issue, which is available at http://wileyonlinelibrary.com.]

Under the assumption that regional covariance in local brain tissue properties reflects anatomical connectivity patterns we focused on investigation of corticosubcortical circuits integrating within the STN. Considering the absence of strong evidence for clear‐cut segregation of STN subregions [Keuken et al., 2012] we use the probabilistic diffusion tractography information only to guide our choice for seeding areas for the structural covariance analysis of quantitative brain tissue property maps. Building on the confirmatory results demonstrating that STN subregions predominantly projecting to limbic and motor areas can be defined using probabilistic diffusion tractography [Lambert et al., 2012], we tested a proof‐of‐concept by looking for anatomically plausible patterns of regional covariance of tissue properties across the brain. To lend face validity of the experiment and reduce effects of a putative interaction between DWI and multiparameter maps, we performed the structural covariance analysis of multiparameter maps in a larger dataset including different participants than those who underwent probabilistic diffusion tractography. On the basis of the structural covariance assumption [Mechelli et al., 2005], we demonstrate a highly plausible anatomical pattern of shared local tissue properties between STN subregions and corresponding motor and limbic basal ganglia areas (Fig. 3). Lastly, the lack of tissue property covariance between subcortical and cortical regions, areas with differential ontogenesis and functional role, can be also seen as additional evidence for the anatomical plausibility of our findings.

#### Motor loop

Local parameter values linked to iron and myelin content within the motor STN subregion showed a positive correlation with the corresponding tissue property maps in the caudal–dorsal putamen and caudal pallidum (Fig. 2). The spatial distribution pattern of anatomical covariance largely overlaps with the anatomical extent of the motor loop delineated in tracing experiments in non‐human primates [Calzavara et al., 2007; Joel and Weiner, 1997; Künzle, 1978; Parent and Hazrati, 1995; Selemon and Goldman‐Rakic, 1985, 1990]. Our results showing a positive correlation of tissue properties between the motor STN and an extended thalamic area going beyond pure motor nuclei most likely reflect similarity owing to spatial proximity rather than connectivity.

#### Limbic/Associative loop

Parameter values in the anterior STN correlated positively with tissue properties in the dorsal and anterior–ventral caudate, including the nucleus accumbens, the anterior putamen, and ventral pallidum (Figs. 2 and 3). Similar to the motor loop findings, the anatomical distribution of basal ganglia covariance of tissue properties with the anteromedial part of the STN is in line with previous findings from animal studies [Haber et al., 2006; Parent and Hazrati, 1995] and imaging data on the reward system in humans [O'Doherty et al., 2004; Pessiglione et al., 2007].

The analysis based on bipartite and tripartite STN shows almost identical covariance patterns. An important distinction, however, is the detected R1 covariance between the associative STN and the dorsal pallidum considered as associative area [Shink et al., 1996]. These findings confirm the tight link between function, anatomical connectivity, and local brain tissue properties of the basal ganglia circuitry.

Even considering the high level of anatomical plausibility of our findings there are methodological limitations of this study to be considered. Led by our intention to provide a framework for optimal delineation of the STN subregions and their corresponding connectivity patterns in other basal ganglia structures our choice of limbic, associative, and motor cortical targets is mainly based on systematic, however potentially subjective selection of evidence from the literature. Together with the current relatively low spatial resolution of the MR images in relation to the STN size, this could explain why the dorsal caudate, receiving important projections from dorsal prefrontal regions, was not found to covary with the associative STN. Considering the known potential pitfalls of the probabilistic diffusion tractography method, we acknowledge the absence of strong evidence for direct connections from STN to hippocampus and amygdala in humans [Groenewegen and Berendse, 1990; Parent and Hazrati, 1995]. Despite the scarce evidence from non‐human primates suggestive for direct projections, part of our tractography findings may stem from erroneous merging of fibers into the uncinate fasciculus creating the impression of monosynaptic projections to amygdala or hippocampus.

In summary, our findings bring strong in vivo evidence for differential tissue properties in limbic and motor STN subregions. The demonstration of regional covariance of myelin and iron content within limbic and motor loops across all basal ganglia structures is a novel finding, which has the potential for practical implementation not only in the target planning phase of STN deep brain stimulation but also as a biomarker for early diagnosis in movement disorders caused by neurodegeneration.

## ACKNOWLEDGMENT

The authors are very grateful to Dr. Thomas Schönecker for his valuable help with defining the anatomical STN borders.
